# In-vitro assessment of the efficiency of cold atmospheric plasma on decontamination of titanium dental implants

**DOI:** 10.1186/s40729-022-00411-9

**Published:** 2022-03-11

**Authors:** Christian Flörke, Josephine Janning, Cedric Hinrichs, Eleonore Behrens, Kim Rouven Liedtke, Sinan Sen, David Christofzik, Jörg Wiltfang, Aydin Gülses

**Affiliations:** 1 Christian Albrechts University, Department of Oral and Maxillofacial Surgery, University Medical Center Schleswig-Holstein, Campus Kiel, Arnold-Heller Straße 3, Haus B, 24105 Kiel, Germany; 2 Christian Albrechts University, Department of Orthopedics, University Medical Center Schleswig-Holstein, Campus Kiel, Kiel, Germany; 3 Christian Albrechts University, Department of Orthodontics, University Medical Center Schleswig-Holstein, Campus Kiel, Kiel, Germany; 4 Christian Albrechts University, Department of Conservative Dentistry, University Medical Center Schleswig-Holstein, Campus Kiel, Kiel, Germany

**Keywords:** Bacteria, Biofilm, Cold atmospheric plasma, Peri-implantitis

## Abstract

**Background:**

The aim of the current study was to comparatively assess the efficiency of three different adjunctive therapy options (cold atmospheric plasma, [CAP], photodynamic therapy [PDT] and chemical decontamination via 35% phosphoric acid gel [PAG]) on decontamination of titanium implant surfaces in-vitro.

**Materials and methods:**

Implants were inserted in concavities of four mm in depth mimicking a bone defect at the implant recipient site. In each model, two implants were inserted in the fourth and one implant in the third quadrants. After contamination with *E. faecalis*, the first group has been treated with CAP for 3 min, the second group with 35% PAG (and the third group with PDT. After treatment, quantification of bacterial colonization was assessed by quantification via colony forming units and qualitatively by fluorescence microscopy and scanning electron microscopy.

**Results:**

With a mean value of 1.24 × 10^5^ CFU/ml, the CAP treated implants have showed the least microorganisms. The highest number of CFU was found after PDT with mean value of 8.28 × 10^6^ CFU/ml. For the implants that were processed with phosphoric acid, a mean value of 3.14 × 10^6^ CFU/ml could be detected. When the groups were compared, only the CAP and PDT groups differed significantly from each other (*p* = 0.005).

**Conclusion:**

A complete cleaning of the micro-textured implant surface or the killing of the bacteria could not be achieved by any of the investigated treatment options, thus bacteria in the microstructure of the titanium surface cannot be completely reached by mechanical and physico-chemical processes.

**Clinical relevance:**

The main goal of the adjunctive peri-implantitis treatment is the decontamination of the implant surface. However, there is still an ongoing need to define the most appropriate adjunctive therapy method. Due to its antimicrobial effects, CAP combined with mechanical debridement could be a feasible treatment modality in the management of peri-implantitis.

## Introduction

Among multitude of available prosthetic treatment options, dental implants have become currently the first-line-treatment protocol in the management of patients suffering from tooth loss. However, despite their high clinical success rates, implant related infections and consequent implant failures remains still challenging for dental clinicians.

Peri-implantitis is a collective term used to describe inflammatory processes in response to a bacterial biofilm that result in the loss of bone around an osseointegrated dental implant. Despite similarities regarding the composition of the bacterial biofilms [1], there are differences in the extent of tissue destruction between peri-implantitis and periodontitis. A peri-implantary bone destruction progresses more rapidly and is usually characterized by crater-shaped bony defects around the implant, which could be explained by the lack of periodontal tissue structures, such as Sharpey's fibers [2]. In addition, it has been reported that peri-implant tissues show reduced vascularization characteristics [3], which might restrict the healing processes of the peri-implant tissue. Therefore, early recognition and proper management of peri-implantitis are predictive factors in the prognosis of the dental implant treatment.

As soon as clinical and/or radiological signs of peri-implantitis have been detected, therapeutic measures, which should address the inflammatory process of the implant area, should be initiated. In the literature, treatment options of peri-implantitis were subdivided basically [4] into non-surgical techniques including power-driven air polishing devices, Er: YAG lasers, metal (e.g., titanium) curettes, and ultrasonic curettes with the plastic sleeve, and surgical management options, such as access surgery, respective surgery or a regenerative procedures. It has been stated that, non‐surgical therapy should always be the first step as this allows the clinician time to evaluate the healing response of the tissues and the patient's ability to perform effective oral hygiene measures [5]. Moreover, it has been proclaimed that, surgical and non-surgical management options should always be combined adjuvant treatment techniques using antiseptic/antibiotic/antimicrobial agents [6], laser-assisted [7], probiotic, photodynamic (PD [8]), and plasma therapies [9] to ensure a decontaminated implant surface; however, the distinctive advantages and definitive benefits of adjuvant therapy options are not proven yet [10].

The main goal of the adjunctive peri-implantitis treatment is the decontamination of the implant surface. However, there is still an ongoing need to define the most appropriate adjunctive therapy method. In recent years, cold atmospheric plasma (CAP) has received increasing attention in dental implantology, due to its antimicrobial effects when combined with mechanical treatment as an adjunctive modality in the management of peri-implantitis [11]. Therefore, the aim of the current experimental study was to assess the efficiency of three different adjunctive therapy options (CAP, PD and chemical decontamination via 35% phosphoric acid gel [PAG]) on decontamination of titanium implant surfaces in-vitro.

## Materials and methods

### Study design

The study group consisted of 45 implants (TiPure Plus BEGO Semados® SC, 3.75 × 8.5 mm, BEGO GmbH & Co. KG, Bremen, Germany) which were divided into 3 equal groups (n:15). Two implants, neither infected nor decontaminated, were excluded and served as negative control. In addition, 2 implants had been preserved non-contaminated and without any treatment. For each group, five lower jaw models (Modell “Lower jaw” Nr: 57464, BEGO GmbH & Co. KG, Bremen, Deutschland) were used. In each model, one implant has been removed prior to decontamination procedure and served as positive control (n_total_:15). Following contamination, implant surfaces were scaled by a blinded dental surgeon for 15 s with a plastic curette (Implacare II tips, Hu-Friedy, Frankfurt am Main, Germany) for biofilm removal. The implants in the first group were decontaminated with CAP, the second group with PAG and the third group with PD, respectively.

### Sample preparation

**Fig. 1:**
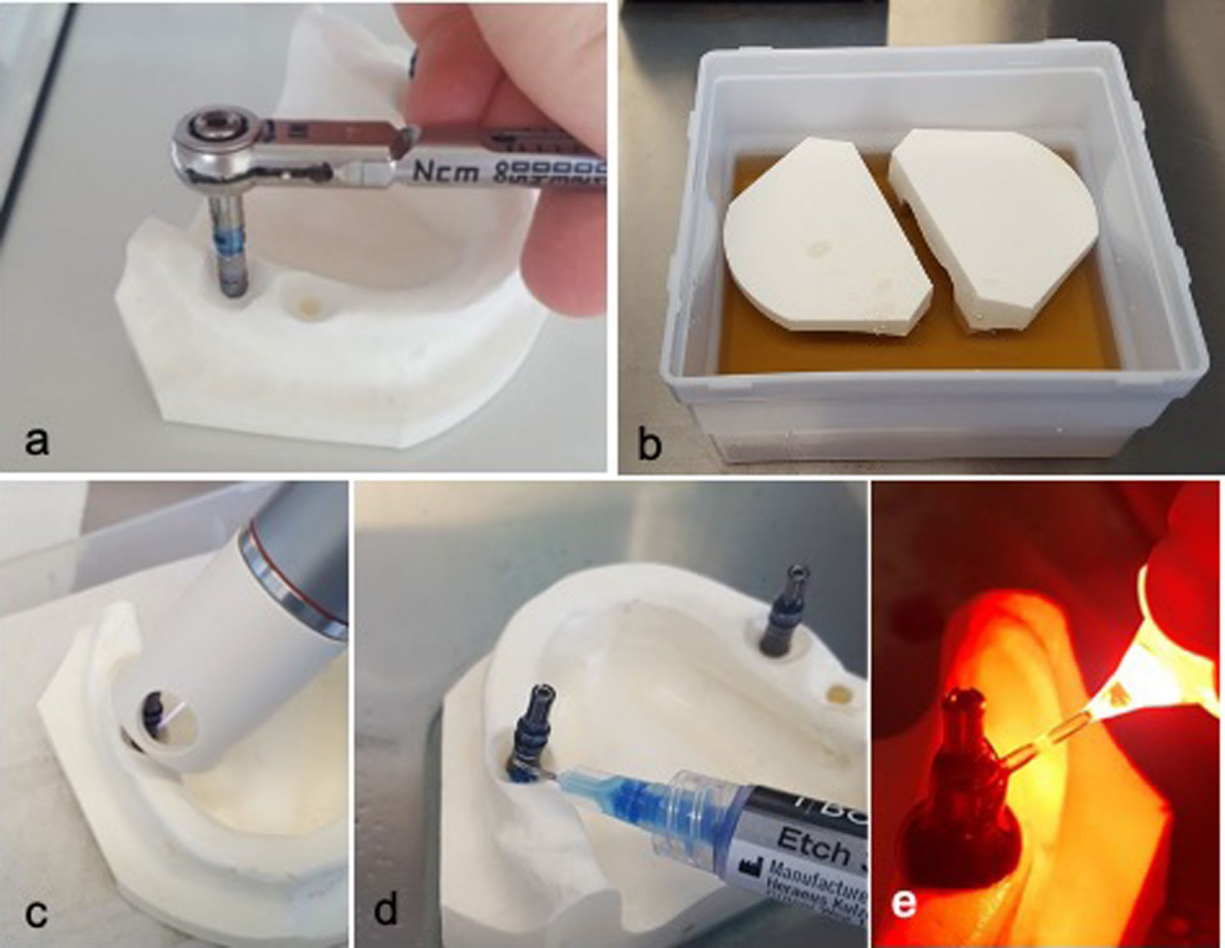
**a**. Prior to preparation of the experimental model—which mimics an in-situ crestal bony defect—implant cavity was prepared according to the manufacturers guidelines and implant was placed following establishment of the experimental defect. **b** Decontamination of the models in bacterial solution **c** Cold plasma application for 3 min using kINPen® MED (neoplas tools GmbH, Greifswald, Germany) with an output of 5 W. The spacer ensured that the correct distance has been maintained. **d** Application of 35% phosphoric acid (iBond etch 35 gel, Heraeus GmbH, Hanau, Germany) to the contaminated implant surfaces. After a contact time of 45 s, the acid was rinsed off with 5 ml of sterile NaCl solution so that no residues remained. **e** The contaminated implant surfaces were treated with photosensitizer toluidine blue (FotoSan®Agent MEDIUM, LOSER & CO GmbH, Leverkusen, Germany) and afterwards with FotoSan 630 LED light pen (LOSER & CO GmbH, Leverkusen, Germany) for 10 s on mesial, distal, lingual, vestibular surfaces with a wavelength of 630 nm using short perio-tips (LOSER & CO GmbH, Leverkusen, Germany)

The implants were inserted according to the manufacturer's drilling protocol with the surgical tray "BEGO Semados® S-Line Tray-Plus". To mimic an in-situ crestal bony defect, a concavity of 4 mm in depth was created at the implant recipient site using a large spherical burr prior to implant insertion. In each model, two implants were inserted in the fourth and one implant in the third quadrants. (Fig. 1a, b).

### Contamination procedure

A cultivation with 10 ml sterile nutrient solution (BHI, Brain–Heart-Infusion Broth, Carl Roth GmbH + Co. KG, Karlsruhe, Germany) and 100 μl bacterial culture with *E. faecalis* (ATCC 29,212) was conducted at 37 °C for 24 h. (Heraeus B6060, Heraeus Holding GmbH, Hanau, Germany). Afterwards, the boxes (Eppendorf pipette tip reusable boxes Eppendorf AG, Hamburg, Germany) containing the models were sterilized at 121 °C (Autoclave Melag Vacuklav 24, MELAG Medizintechnik oHG, Berlin, Germany). The models with implants were placed in the boxes with the implants facing towards the bottom of the box, to enable continuous wetting with bacterial suspension. On the first day, the implants were infected with 200 ml of sterile BHI and 100 μl of the overnight culture and then incubated at 37 °C (Scientific C24 Incubator Shaker, New Brunswick Scientific, Edison, New Jersey, USA). After 4 h, the optical density was controlled via spectrophotometer (BioPhotometer 6131, Eppendorf AG, Hamburg, Germany) at 600 nm (OD600), which was set to 0.8. Nutrient solution was exchanged in every 24 h with 200 ml of sterile BHI for 6 days.

### Mechanical debridement

Prior to adjuvant treatment, all implant surfaces were first curretted for 15 s with a plastic curette (Implacare II tips, Hu-Friedy, Frankfurt am Main, Germany), which was applied at a recommended working angle by a single researcher who was blinded to the study.

### CAP treatment

Each implant in the first group has been treated with cold plasma for 3 min using kINPen® MED (neoplas tools GmbH, Greifswald, Germany) with an output of 5 W. The spacer ensured that the correct distance has been maintained (Fig. 1c).

### PAG treatment

35% phosphoric acid (iBond etch 35 gel, Heraeus GmbH, Hanau, Germany) was applied to the contaminated implant surfaces. After a contact time of 45 s, the acid was rinsed off with 5 ml of sterile NaCl solution so that no residues remained (Fig. 1d).

### PDT

The contaminated implant surfaces were treated with photosensitizer toluidine blue (FotoSan®Agent MEDIUM, LOSER & CO GmbH, Leverkusen, Germany) and afterwards with FotoSan 630 LED light pen (LOSER & CO GmbH, Leverkusen, Germany) for 10 s on mesial, distal, lingual, vestibular surfaces with a wavelength of 630 nm using short perio-tips (LOSER & CO GmbH, Leverkusen, Germany) (Fig. 1e). To completely remove the dye from the implant surface after treatment, the implant surfaces were rinsed off with 5 ml of sterile NaCl solution.

### Colony forming units (CFU)

**Fig. 2:**
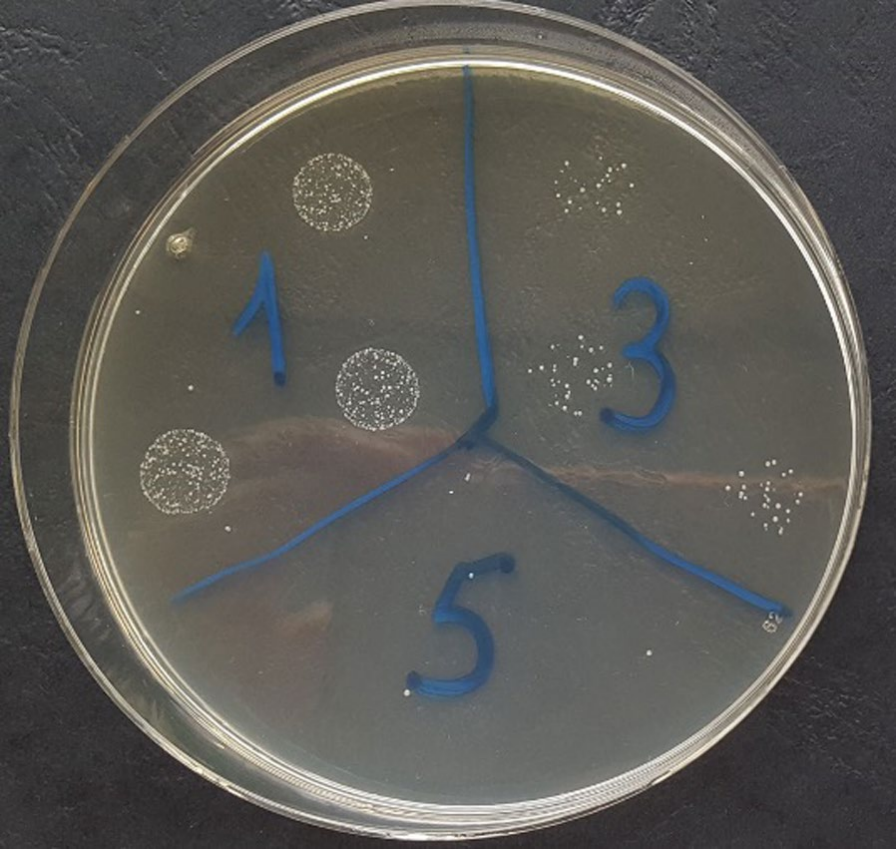
Caso agar plates (CASO-Agar Ph.Eur., Carl Roth GmbH + Co. KG, Karlsruhe, Germany) were placed in an incubator at 37 °C (Heraeus B6060 incubator, Heraeus Holding GmbH, Hanau, Germany). After 24 h, the colony-forming units were counted with the germ counter

After removal, each implant was placed in an Eppendorf tube containing 1 ml of sterile NaCl solution. To de-attach the bacteria from the implant surface, the implants were placed in an ultrasonic bath (ultrasonic bath Branson 2210R-MT Ultrasonic Cleaner, Branson Ultrasonics Corporation, Danbury/CT, USA) for 20 min. The bacterial suspension was then diluted to 10^–2^ and later to 10^–4^. Afterwards, the different dilution levels were applied on Caso agar plates. These Caso agar plates (CASO-Agar Ph.Eur., Carl Roth GmbH + Co. KG, Karlsruhe, Germany) were placed in an incubator at 37 °C (Heraeus B6060 incubator, Heraeus Holding GmbH, Hanau, Germany). After 24 h, the colony-forming units were counted with the germ counter (Germ counter BZG 25 from WTW, Xylem Analytics Germany Sales GmbH & Co. KG, Weilheim, Germany) (Fig. 2).

### Fluorescence microscopy

The implants were carefully removed from the Eppendorf tubes following ultrasonic bath. The suspensions were centrifuged for 10 min with a relative centrifugal acceleration (RCF) of 3000 and a temperature of 20 °C (centrifuge Sigma 3K30, rotor 12154, Sigma Laborzentrifugen GmbH, Osterode am Harz, Germany). The supernatant was then carefully sucked off so that 50 μl remained in the tube. 50 μl of the LIVE/DEAD BacLight staining solution (LIVE/DEAD Baclight Bacterial Viability Kit L7007 (Molecular Probes, Inc., Eugene, Oregon, USA) has been prepared and added to each Eppendorf tube. The tubes were then placed on the vortex mixer (vortex mixer K-550-GE, Bender & Hobein AG, Zurich, Switzerland) for 10 s and then incubated at room temperature and in the dark environment for 15 min. Afterwards, 5 μl from each reaction tube were applied to a microscope slide and covered with a cover slip and immersion oil. To prevent the fluorescent dyes from bleaching, the respective slide was only prepared directly before the microscopic examination. In the meantime, the reaction vessels were stored in the dark. The Axioplan 2 microscope with the AxioCam MRc digital camera and the AxioVision software package (all from Carl Zeiss Microscopy GmbH, Jena, Germany) were used for the evaluation. 10 random image sections were selected from each implant, which were recorded with the emission filter 470/40 for green bacteria and once with the emission filter 546/12 for red bacteria at a magnification of ×100, respectively.

### Scanning electron microscope

The implants were washed for 1 min with PBS (phosphate-buffered saline solution, Dulbeco, Biochrom GmbH, Berlin, Germany) and later with 1 ml of 4% glutaraldehyde. Afterwards, the implants were washed three times for 5 min each time with PBS. The dehydration was carried out by means of an ascending series of 30%, 50%, 70%, 90% and 100% ethanol. The implants were then air-dried until ethanol had completely evaporated. The implants were then attached to SEM sample plates (Agar Scientific Ltd, Stansted, Essex, United Kingdom) and stored overnight in a desiccator (Erich Eydam KG, Kiel, Germany). Gold sputtering with a thickness of 5 nm (BAL-TEC SCD 500, Leica Microsystems GmbH, Wetzlar, Germany) and examination using a scanning electron microscope (Philips XL 30 ESEM, Philips GmbH Market DACH, Hamburg, Germany) has been performed. For each implant, five corresponding area at a magnification of 5000 and five area at a magnification of 8000 were recorded. A semi-quantitative evaluation was performed as described by Acil et al. [12]. According to that, classification of colonization was classified as: (a) grade 1: no microorganisms detectable; (b) grade 2: sparse microorganisms; (c) grade 3: many microorganisms and conglomerates.

### Statistical analysis

Descriptive statistical analysis was carried out using IBM, SPSS, Statistics version 24.0 for Windows (SPSS Inc., Chicago, IL, USA). The non-parametric Kruskall–Wallis and post-hoc tests were used to analyze the CFU values, LIVE/DEAD BacLight quotients and the semi-quantitative evaluation of the SEM-images. To compare the fluorescence microscopic data, the one-way analysis of variance (ANOVA) and subsequent post-hoc tests were carried out. A significance level was set to *p* < 0.05.

## Results

Gram staining revealed that all bacterial suspensions showed the presence of chain-formed gram-positive cocci.

### CFU

With a mean value of 1.24 × 10^5^ CFU/ml, the CAP treated implants have showed the least microorganisms. The highest number of CFU was found after PDT with mean value of 8.28 × 10^6^ CFU/ml. For the implants that were processed with phosphoric acid, a mean value of 3.14 × 10^6^ CFU/ml could be detected (Table 1).

**Table 1:** CFU values regarding the subgroups

Subgroup	*M* [CFU/ml]	Md [CFU/ml]	Min [CFU/ml]	Max [CFU/ml]	SD [CFU/ml]
Control (+)	7.12 × 10^6^	4.7 × 10^6^	2.9 × 10^6^	1.6 × 10^7^	4.7 × 10^6^
CAP	1.24 × 10^5^	1.1 × 10^5^	1.0 × 10^5^	1.8 × 10^5^	3.36 × 10^4^
PAG	3.14 × 10^6^	2.1 × 10^6^	1.5 × 10^6^	6.3 × 10^6^	2 × 10^6^
PDT	8.28 × 10^6^	7.1 × 10^6^	3.5 × 10^6^	1.6 × 10^7^	4.9 × 10^6^

**Fig. 3:**
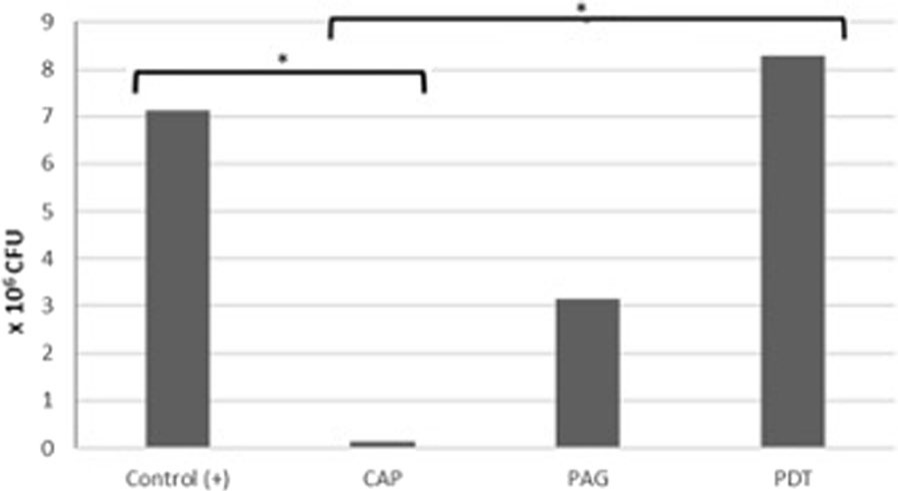
Significantly fewer colony-forming units could develop on the agar plates of the plasma-treated implants than on the positive controls (*p* = 0.006). The implants treated with PDT (*p* = 1.000) and those treated with PAG (*p* = 0.874), on the other hand, showed no significant difference to the positive controls. When the groups were compared, only the CAP and PDT groups differed significantly from each other (*p* = 0.005). **

The Kruskall–Wallis test and subsequent post-hoc tests (Dunn–Bonferroni tests) were used to determine the differences between subgroups. It could be shown that significantly fewer colony-forming units could develop on the agar plates of the plasma-treated implants than on the positive controls (*p* = 0.006). The implants treated with PDT (*p* = 1.000) and those treated with PAG (*p* = 0.874), on the other hand, showed no significant difference to the positive controls. When the groups were compared, only the CAP and PDT groups differed significantly from each other (*p* = 0.005). (Fig. 3).

### Number of live microorganisms

**Fig. 4:**
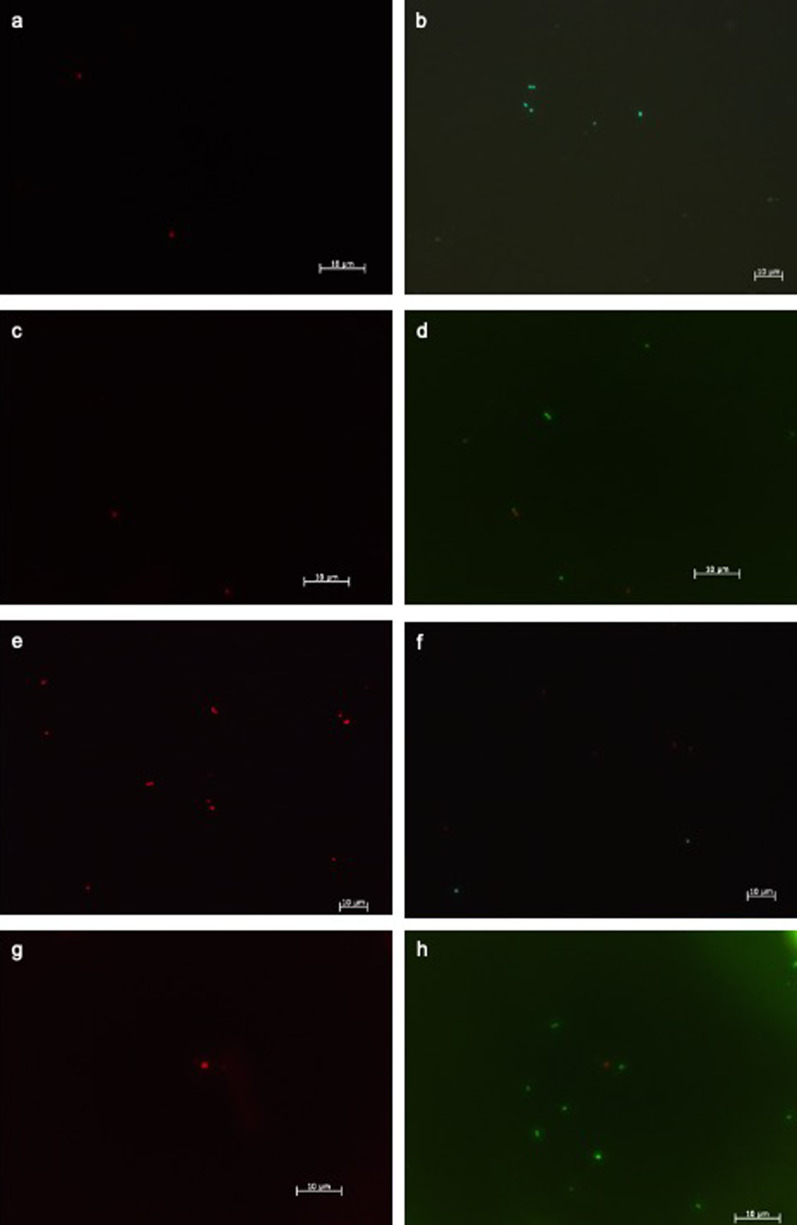
Fluorescence microscope image with the emission filter 470/40: green/living bacteria showing **a**, **c**, **e** and **g** for positivecontrol, PDT, plasma and phosphoric acid subgroups, respectively, and emission filter 546/12 showing red/dead bacteria in **b**, **d**, **f** and **h** for positive control, PDT, plasma and phosphoric acid subgroups, respectively. All groups were demonstrated in a 100-fold magnification

**Fig. 5:**
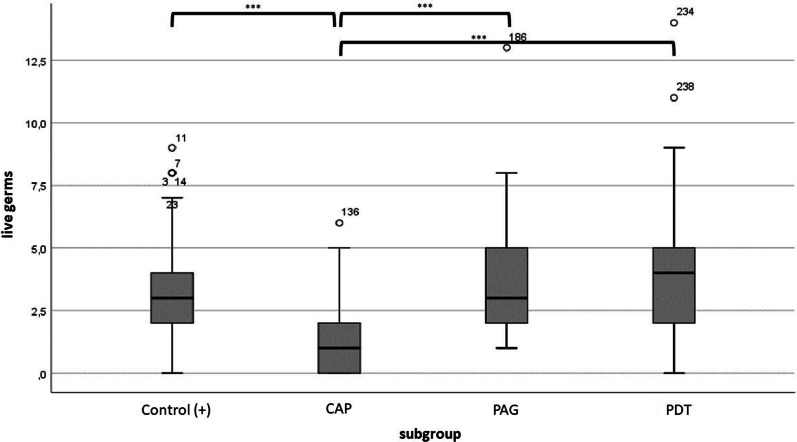
Box plot representation of the number of living germs per image section in the DEAD/LIVE BacLight fluorescence staining and significant differences (***p ≤ 0.001); positive: positive-control group;  with case number: slight outliers (more than 1.5 standard deviations from the mean). Please note the difference between CAP and other subgroups

Figures 4 and 5 clearly show that the lowest average live microorganisms can be assigned to the plasma treatment. This means that a relatively large number of germs were killed after plasma therapy.

After plasma therapy, only 1.28 germs per image section survived. Phosphoric acid and PDT in turn left an average of 3.74 and 4.24 living germs after the therapy. After using the one-way ANOVA and subsequent post-hoc tests (Tukey tests), a significant difference can be found in the plasma group compared to the other two therapy options (*p* < 0.001), whereas the phosphoric acid group and the PDT group do not differ significantly to each other (*p* = 0.699).

If the positive controls were also assessed, it can be stated that significantly fewer living germs were present after plasma therapy than after no treatment (*p* < 0.001). With phosphoric acid therapy and PDT, even more living germs were found on average than with the positive control. However, there was no statistically significant difference.

In addition, red–green quotient was calculated by dividing the number of red germs by those of green germs. The higher this value, the greater the proportion of dead germs, and consequently the better the decontamination performance of the respective therapy option could be established. The phosphoric acid therapy showed a significantly lower value with an average red–green quotient of 0.9, as did the PDT with an average red–green quotient of 0.8. Thus, the red–green quotients of phosphoric acid and PDT show a significant difference to the red–green quotient of the plasma group and thus produced significantly fewer dead germs than plasma therapy. The positive controls showed, with a small average red–green quotient of 0.4, that a small number of red and many green germs were counted. It also became clear that both the red–green quotient of the phosphoric acid therapy and the red–green quotient of the PDT did not differ significantly from the positive controls, in contrast to the red–green quotient of the plasma treatment. This in turn differs significantly from the quotient of the positive controls Descriptive statistics of dead bacteria revealed that, with an average of 5.32 red bacteria per image section, significantly higher number of dead germs was obtained after CAP treatment, followed by PDT (3.40) and PAG (3.30) (*p* = 0.001). Compared to the positive controls, there was a significant difference following all three treatment options (*p* < 0.001) (Table 2).

**Table 2:** After plasma therapy, only 1.28 germs per image section survived

	Mean	Median	Minimum	Maximum	SD
Control (+)	3.54	3.00	0.00	13.00	2.38
CAP	1.28	1.00	0.00	6.00	1.6
PAG	3.74	3.00	1.00	13.00	2.35
PDT	4.24	4.00	14.00	8.00	2.68

Phosphoric acid and PDT in turn left an average of 3.74 and 4.24 living germs after the therapy

After using the one-way ANOVA and subsequent post-hoc tests (Tukey tests), a significant difference can be found in the plasma group compared to the other two therapy options (*p* < 0.001), whereas the phosphoric acid group and the PDT group do not differ significantly to each other (*p* = 0.699)

### *Semi-quantitative assessment *via* SEM*

#### Positive control

**Fig. 6:**
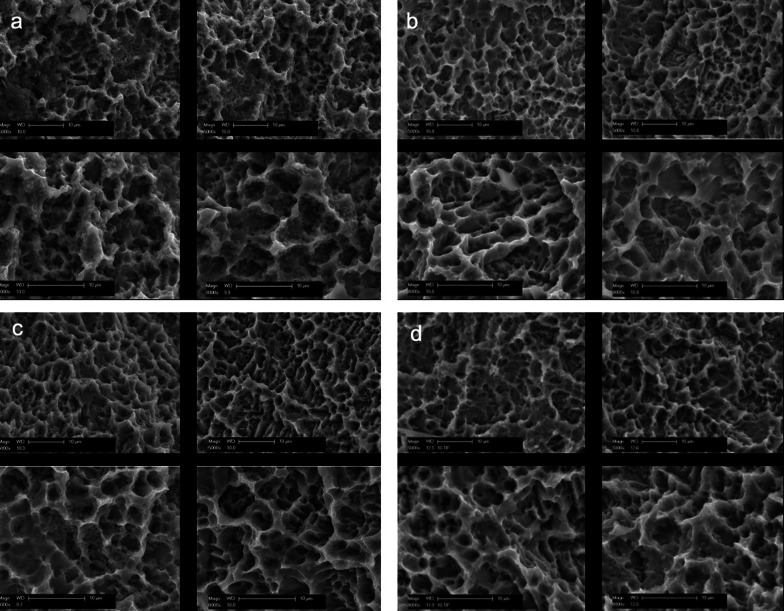
**a** SEM images of the implant surfaces of the positive controls with *E. faecalis*; top left and top right: 5000× magnification; bottom left and bottom right: 8000 × magnification. All untreated samples who served as control showed clear microbial growth on the sandblasted and acid-etched implant surface under the scanning electron microscope. The specific form of *E. faecalis* could also be identified. **b** SEM images of plasma-treated implant surfaces; top left and top right: 5000× magnification; bottom left and bottom right: 8000× magnification. A significantly reduced number of bacteria could be observed. **c** SEM images of phosphoric acid applied implant surfaces; top left and top right: 5000× magnification; bottom left and bottom right: 8000× magnification. Particularly a reduced number of bacteria could be observed. **d** SEM images of PDT applied implant surfaces; top left and top right: 5000× magnification; bottom left and bottom right: 8000× magnification. Despite reduced number of bacteria compared to the positive controls, large amounts of germs could be found

All untreated samples who served as control showed clear microbial growth on the sandblasted and acid-etched implant surface under the scanning electron microscope (Fig. 6a).

#### CAP

A significantly reduced number of bacteria was found on the implants after mechanical and plasma treatment compared to the positive controls, which was classified as “ + ” in 94% of the samples (*n*:47). A complete decontamination of the titanium surface could be seen in very few image sections (*n*:2) (Fig. 6b).

#### PAG

Implants processed with phosphoric acid after mechanical treatment with plastic curette showed a visible decrease in the number of bacteria under the SEM compared to the positive control samples. Nevertheless, a high level of germs could be observed particularly. These accumulations of bacteria remained in the notches and furrows of the titanium surface. The surface was neither damaged nor changed (Fig. 6c).

#### PDT

The number of bacteria was reduced by the therapy with plastic curette and PDT compared to the positive controls (Fig. 6d). However, large amounts of germs could be found in some image sections, which are not only collected in the undercuts and depressions of the implant surfaces.

**Fig. 7:**
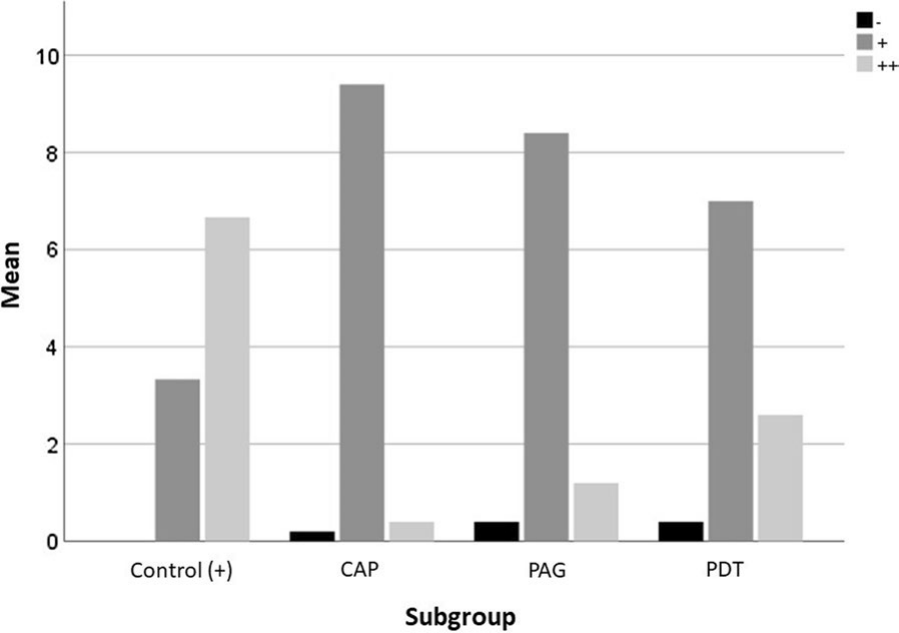
Distribution of the semiquantitative bacterial colonization scores regarding the subgroups;—(no visible bacteria), + (isolated/few bacteria), ++ (many bacteria)

The semi-quantitative evaluation of the subgroups showed that 67% of the image sections could be assigned to the ++ level with the highest microbial growth and 33% to the + level (Table 3 and Fig. 7). No image section showed completely bacteria-free areas.

**Table 3:** Semi-quantitative evaluation of the subgroups showed that, 67% of the image sections could be assigned to the ++ level with the highest microbial growth, whereas 33% to the + level in the control group

Subgroup	−		+		++		Total	
Control (+)	0	0%	20	33%	40	67%	60	100%
CAP	1	2%	47	94%	2	4%	50	100%
PAG	2	4%	42	84%	6	12%	50	100%
PDT	2	4%	35	70%	13	26%	50	100%

Low colonization has been mostly detected in the CAP group

Overall, none of the three therapy methods could achieve a complete elimination of bacteria from the entire implant surface. The proportion of image sections with no bacteria at all (−) was comparably low in all three groups. However, PDT group showed a particularly high proportion of fields of vision in which many bacteria could still be seen. PAG had about half as many excerpts that were rated ++ . The plasma group recorded the fewest fields with very many bacteria. The number of fields rated ++ (*p* = 0.016) and + (*p* = 0.009) showed to be of significant difference to the positive-control in the plasma group, whereas no significant difference could be found in the PDT or PAG groups.

Furthermore, it was found that none of the three therapeutic approaches led to changes or damage to the titanium surface.

## Discussion

The aim of the present work was to investigate the effectiveness of different therapy methods for the surface decontamination of titanium implants for the treatment of peri-implantitis. For this purpose, plasma treatment as a novel cleaning method was compared with the two established options. Overall, it could be shown that plasma is a promising method for cleaning titanium implants. In addition, the establishment of a test model with *E. faecalis* was also successful.

Treatment with cold plasma for 3 min per implant and an output of 5 W turned out to be the most effective method for decontaminating titanium implants in this experiment. Both the CFU and the live-dead fluorescence staining showed a significant difference between the plasma and positive controls. 1.24 × 10^5^ CFU/ml could be detected on the plasma-treated samples. In comparison, the untreated positive samples with 7.12 × 10^6^ CFU/ml had significantly more colony-forming units per ml. The live-dead fluorescence staining showed that with an average of 5.32 red germs per image section, the greatest number of dead germs could be achieved by plasma treatment. No treatment (positive control) resulted in an average of 1.29 red germs per image section. With regard to the high red–green ratio of 4.2, it becomes clear that plasma produced the best contamination performance in this study. The semi-quantitative evaluation of the SEM images also led to the result that the plasma therapy led to the greatest reduction in bacteria compared to the other two investigated methods.

Within the last two decades, the antibacterial effect of the plasma has already been proven in numerous studies [13–18] showed that plasma is also able to lead to a reduction in the number of living bacteria on the micro-structured surface of dental titanium implants. Compared to the diode laser, plasma had the ability to penetrate into porous structures and thus effectively disinfect the complex three-dimensional surface [19]. Also confirmed that biofilm on micro-structured titanium discs could be inactivated by means of plasma without changing the surface. However, complete removal of the biofilm could only be achieved with the additional use of an air–water spray.

The role of PDT in peri-implant therapy has not yet been clearly established. Both in vitro and in vivo tests showed different results, which do not yet allow any clear conclusions. Some clinical studies could not find any differences between mechanical cleaning of the implants alone and the additional use of PDT [20–22]. The clinical effect of PDT, therefore, remains questionable.

Eick et al. showed based on their in vitro study that PDT with toluidine blue and an LED in the red spectrum (wavelength 625–635 nm) are able to reduce the viability of pathogenic germs [23]. Doertbudak et al. previously were also able to show that the use of toluidine blue and laser on titanium implants led to a significant reduction in all three examined bacterial species [24]. However, neither Eick et al. nor Dörtbudak et al. could show that the biofilm could be completely destroyed.

The effectiveness of PDT against *E. faecalis* was investigated particularly in connection with the disinfection of root canals, since *E. faecalis* is often associated with persistent endodontic infections [25].

In the current study, the decontamination performance of PDT on a biofilm from *E. faecalis* that has settled on a microstructured surface was examined, which might be a reason for the less successful results of PDT [26]. We´re also able to show in their investigations that with the help of PDT, individual cells or monolayers could be successfully eliminated compared to biofilms. In addition, PDT had a significantly lower antibacterial effect on *E. faecalis* than Streptococcus anginosus and Fusobacterium nucleatum. The higher resistance of *E. faecalis* to PDT could be attributed to the fact that *E. faecalis* has the ability to independently produce oxygen radicals [27]. Since the antibacterial effect of PDT is due to these oxygen radicals, this could also be a possible cause for the relatively weak antibacterial effect of PDT against *E. faecalis*.

The effectiveness of phosphoric acid for bacterial decontamination of microstructured titanium discs has already been demonstrated by Dostie et al. [28]. The results showed that after treatment with phosphoric acid, living bacteria remained in the depressions of the titanium surface and that comparable cleaning could be achieved by simply rinsing with sterile saline solution. The ability to remove bacteria, therefore, appeared to be based solely on the mechanical action of the rinsing, rather than a chemical action of the phosphoric acid. In clinical studies, too, the use of phosphoric acid showed an effect similar to that of the mechanical treatment of the implants [29].

The aim of this experimental study was to comparatively assess the decontamination efficiency of three different methods which were mainly used in the management of peri-implantitis as an adjuvant therapy option. Despite underlying possible clinical aspects and expectations, current experimental study model could mimic whether peri-implantary bone defect secondary to peri-implantitis, nor a multibacterial condition, regarding the experimental mono-microbial contamination design, which differs from the multi-bacterial clinical condition. Besides that, for experimental studies focusing on peri-implantitis, mono-bacterial contamination design could be a more reliable method in terms of standardization of the bacterial colonization [30]. Therefore, current mono-bacterial experimental model has been selected to improve the quantification of the results.

The current study was based on a mono-bacterial contamination model to standardize the contamination and colonization formation. However, it has also been shown that gram-negative bacteria, due to their thinner cell wall, are more quickly deactivated by plasma than gram-positive bacteria with a thicker cell wall [31]. As a mixed culture with gram-negative bacteria, a better response of the gram-positive germ to the plasma therapy could be achieved. It is believed that a change in the extracellular matrix is the cause of this phenomenon. These observations mean that the results presented here have to be interpreted with regard to the differences between gram-positive and gram-negative bacteria.

It is well-known that there are differences in the resistance behavior between biofilms from monocultures compared to biofilms from mixed cultures, for example with regard to disinfectants [32]. Since a pure monoculture of the gram-positive bacterium *E. faecalis* was used in this study, the results obtained here cannot be fully transferred to the clinical picture of peri-implantitis, thus a heterogeneous biofilm of both gram-positive and gram-negative species is involved in this infection.

## Conclusion

Overall, both PDT and phosphoric acid treatment have the ability to reduce bacteria, whereas PDT has shown the least bactericidal ability. Compared to cleaning the implants with plasma, are, therefore, less effective in terms of bacterial elimination.

A complete cleaning of the micro-textured implant surface or the killing of the bacteria could not be achieved by any of the investigated treatment options, thus bacteria in the microstructure of the titanium surface cannot be completely reached by mechanical and physico-chemical processes.

## Data Availability

All data are available in the archive of the Research Laboratories, Christian Albrechts University, Department of Oral and Maxillofacial Surgery.

## Abbreviations

CAP: Cold atmospheric plasma
CFU: Colony forming units
PAG: Phosphoric acid gel
PDT: Photo-dynamic-therapy
SEM: Scanning electron microscope
USA: United States of America
